# Assessing Health Risk due to Exposure to Arsenic in Drinking Water in Hanam Province, Vietnam

**DOI:** 10.3390/ijerph110807575

**Published:** 2014-07-24

**Authors:** Tung Bui Huy, Tran Thi Tuyet-Hanh, Richard Johnston, Hung Nguyen-Viet

**Affiliations:** 1 Center for Public Health and Ecosystem Research (CENPHER), Hanoi School of Public Health, Hanoi, 138 Giang Vo Street, Hanoi, Vietnam; E-Mails: kdtc.hn@gmail.com (T.B.H.); tth2@hsph.edu.vn (T.T.T.-H.); 2 Hanoi Medical College, 35 Doan Thi Diem Street, Hanoi, Vietnam; 3 Department of Environmental Health, Hanoi School of Public Health, 138 Giang Vo Street, Hanoi, Vietnam; 4 Sandec-Department of Water and Sanitation in Developing Countries, Swiss Federal Institute of Aquatic Science and Technology (EAWAG), Ueberlandstrass 133, CH-8600 Dübendorf, Switzerland; E-Mail: johnstonr@who.int; 5 Swiss Tropical and Public Health Institute (Swiss TPH), Socinstrasse 57, CH-4002 Basel, Switzerland; 6 International Livestock Research Institute (ILRI), Hanoi, Vietnam

**Keywords:** Arsenic, drinking water, skin cancer risk, environmental health risk assessment, Vietnam

## Abstract

We assessed health risks related to Arsenic (As) in contaminated drinking water in Hanam, applying the Australian Environmental Health Risk Assessment Framework, which promotes stakeholder involvement in risk assessments. As concentrations in 300 tube-well water samples, before and after filtration, were analyzed and the water consumption levels in 150 households were estimated. Skin cancer risk was characterized using Cancer Slope Factor index and lifetime average daily dose with a probabilistic approach. The results showed that arsenic concentrations in tube-well water ranged from 8–579 ppb (mean 301 ppb) before filtration and current sand filters used by the households did not meet the standard for As removal. Arsenic daily consumption of 40% of the adults exceeded the level of TDI (Tolerable Daily Intake) at 1 µg/kg/day. The average skin cancer risk in adults due to consuming filtered tube-well water for drinking purpose were 25.3 × 10^−5^ (using only well water) and 7.6 × 10^−5^ (using both well and rain water). The skin cancer risk would be 11.5 times higher if the water was not filtered. Improvement of filtration measures or the replacement of the current drinking water sources to minimize the health risks to the local population is urgently needed.

## 1. Introduction

Arsenic contamination in drinking water is of great concern for public health because of its effects on human health. In addition to acute poisoning, arsenic causes chronic health effects, including cancer (skin, lung, and bladder cancer, among others) and non-cancer end points (skin diseases and effects on the cardiovascular, pulmonary, nervous, endocrine, and reproductive systems) [1,2]. Researchers in the last decades have shown that the risks of having cancers were higher among people who had regular exposure to high arsenic concentrations in the environment [3,4]. This is similar to the findings from Bangladesh, India, and Nepal [5,6,7,8,9]. Arsenic in drinking water is a severe public health risk in Vietnam where UNICEF estimates there are approximately 10–15 million people (about 13.5% of the population) using drinking water from tube wells. Studies have shown that arsenic contamination in groundwater is concentrated in a number of provinces in the Red River Delta, Northern Vietnam [10].

From 1995 to 2000, several research projects investigated the source of arsenic in the groundwater, pollution levels and shipping cycle, and found arsenic concentrations in water samples in the areas of Son La, Phu Tho, Bac Giang, Hai Phong, Thai Binh, Nam Dinh, and Thanh Hoa exceeded international and Vietnamese acceptable standard levels for arsenic in drinking water [11]. From November 2003 to April 2004, a large-scale survey in 12 provinces with 12,461 well-water samples showed that the Red River basin provinces, including Ha Nam, Nam Dinh, Ha Tay, Hung Yen, and Hai Duong, were contaminated with arsenic at levels from mild to severe. This suggested that residents living in the Red River Delta area, who use drill well water for drinking purposes, are also at risk of arsenic poisoning with similar levels in Bangladesh [12]. However, the risk has not been specifically quantified. According to a study published in 2010, approximately seven million people in the Red River Delta region use ground water contaminated with arsenic, manganese, selenium, and barium. The levels of arsenic contamination in many locations surveyed in Ha Nam and Hung Yen were found to be as high as in Bangladesh [13].

Hanam province—60 km from South Hanoi—has been facing serious arsenic contamination in groundwater, and Chuyen Ngoai Commune, Duy Tien District, has been identified as an arsenic hotspot in the province. Tube wells serve as the main source of drinking water for local residents. Many people have the habit of using rainwater for cooking and drinking in the rainy season, but due to economic conditions and the space requirement to build large water tanks, people also use water from wells for drinking. Most of the households use domestic sand filters to treat groundwater before drinking, to remove iron and odors. These simple sand filters also remove arsenic, with an average reduction rate of 80% [14]. Nonetheless, filtered water may still well exceed health-based guidelines for arsenic. This study applies a Quantitative Health Risk Assessment approach [15], based on the Australian Environmental Health Assessment framework, and is the first study in Vietnam using a risk assessment approach to determine the health burden due to arsenic exposure in drinking water, and the impact of sand filtration on reducing this burden.

## 2. Methods

### 2.1. Study Site

**Figure 1:**
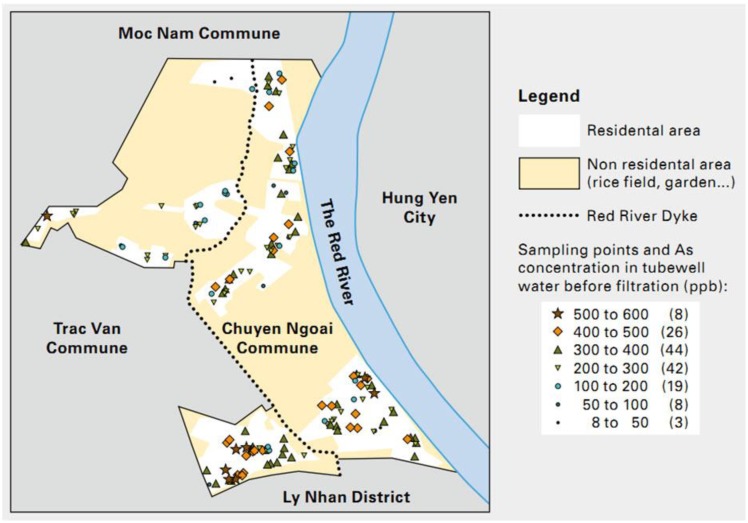
Sampling site and As contamination (before filtration) in the Chuyen Ngoai Commune.

We conducted this study in Chuyen Ngoai Commune, located at the east of Duy Tien District, Hanam Province. 60% of the Commune is outside the Red River dike. The natural land area of the Commune is 877.67 ha, in which the arable land area is 508.42 ha (Figure 1). There is a population of 9324 people, living in 2347 households. People living inside the Red River dike account for 45% of the population. The population living outside the dike (55%) is frequently threatened by floods. Chuyen Ngoai Commune was identified as a hot spot of arsenic contamination in groundwater, but as of yet, no study has been done to assess the health risks to the people living there.

### 2.2. Environmental Health Risk Assessment

We applied the four main steps of the Australian Environmental Health Risk Assessment (EHRA) Framework to assess the health risks related to exposure to As contaminated drinking water, this framework was specifically developed for assessing health risks from environmental hazards [15]. Although there are many similar risk assessment frameworks, we used the Australian framework as it has a clear and important stakeholder involvement, increasing the participation rates of the stakeholders. The four steps are briefly described below.

#### 2.2.1. Issue Identification and Hazard Identification

Health problems affecting people in Chuyen Ngoai Commune from exposure to arsenic in tube wells for drinking water were identified. This was done through the collection and analysis of available data, and interviews with experts in the field of environmental health, particularly with people having experience in arsenic pollution in water. Information about the study area, including geographic locations, socio-economic characteristics, population structure, and previous environmental health research conducted in Chuyen Ngoai Commune, was collected and analyzed. Fourteen water samples were randomly taken at different locations in the Commune to do a quick arsenic test in the field, using the Merck rapid analysis kits. Preliminary results from the analysis of the environmental samples were used to determine whether a full environmental health risk assessment study should be done.

#### 2.2.2. Dose-Response Assessment

Data on arsenic toxicology were reviewed to determine the relationship between different levels of exposure and health effects. Information about Lethal dose 50%–LD50, Minimum Risk Level–MRL, No Observed Adverse Effect Level–NOAEL, Lowest Observed Adverse Effect Level–LOAEL, and Tolerable Daily Intake–TDI were reviewed and summarized.

#### 2.2.3. Exposure Assessment

The purpose of the exposure assessment was to estimate to what extent the population in Chuyen Ngoai Commune was exposed to As through drinking As contaminated water. 150 households were randomly selected from all 2347 households on the list provided by the Communal People’s Committee. This list comprised only households having tube-wells. The sample size was calculated based on a 90% expected proportion of household use of tube well water (based on a local survey of the Department of Health of Hanam province), a precision of 5% and a 95% confidence level. All household members above 12 months of age were eligible. The sampling started by selecting an element from the list of all 2347 households at random and then every *k*th element in the sampling frame was selected until we reached the sample size of 150 households, where k, the sampling interval was calculated as k = N/n; where *n* was the sample size (150 households), and *N* is the population size (2347 households), so the k value was 16.

Each household’s representative answered a questionnaire on general household information, quantity of drinking water, and practices regarding water consumption for drinking and other domestic purposes. The types of water sources and any water treatment currently applied were also recorded. Interviewers collected water samples for arsenic analysis from the tube-wells and the outlet of domestic sand filters. Therefore, in each household, two water samples (before and after filtration) were collected. For water before filtration, wells were pumped for 5–10 minutes before collecting samples. Filtered water from the tank was directly collected without a waiting time. All samples were filtered in the field (0.45 µm cellulose acetate membrane, Sartorius) and stored in acid-washed PVC bottles and acidified to pH < 2 using HNO_3_ [16,17]. Water samples were analyzed for arsenic, using hydride generation and flame atomic absorption spectroscopy (Shimadzu AA-6800). All tests were completed within one month from the sampling date. Quality control tests were made after every five samples.

**Estimation of TDI:** the TDI of each age group *i* in the study population was estimated as follows:

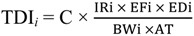

Where **TDI*i*** is Tolerable Daily Intake of group *i*, C represents the arsenic concentration in the drinking water source (in mg/L), **IR_i_** is the intake rate, or the amount of drinking water ingested (L/day), **EF_i_** is the exposure frequency (days/year that the arsenic-contaminated source is used), and **ED_i_** is the exposure duration (years of using the contaminated source). **BW_i_** is the average body weight (kg) for the age class, and **AT** is the life expectancy (days). **AT** was taken as 25,550 days, reflecting a life expectancy of 70 years in Vietnam [18]. (We used four groups of ages including <2 years, 2–6 years, 6–16 years, and 16 years. The IR parameter was taken from the literature, as shown in Table 1.

#### 2.2.4. Risk Characterization

We characterized the risk by (i) comparing As concentrations and TDI with the guideline values, and (ii) estimating the risk of cancer using the Cancer Slope Factor index and lifetime average daily dose with a probabilistic approach. Arsenic is also known to cause internal cancers (notably bladder and lung cancer), but the EPA’s IRIS database does not provide slope factors for these endpoints. In this study, only skin cancer risk was considered because of the availability of CSF.

For risk characterizations using the Cancer Slop Factor, for each age group *i*, the risk of cancer due to arsenic exposure was calculated using Equation 1 [19]:

Risk = TDI × SF × ADAFi
(1)

The **SF** was taken as 1.5 (mg/kg·day)^−1^, reflecting the dose-response relationship for non-melanoma skin cancer [20]. The age-dependent adjustment factor (ADAF) is taken from the literature, and is shown in Table 1.

**Table 1:** Age-specific model parameters.

Parameter	<2 years	2–6 years	6–16 years	>16 years	Source
IR, L/day	1	1	2	2	[21]
BW, kg	10.5	18.2	27.5	50.5	[22,23]
ADAF	10	3	3	1	[19]

The following scenarios were examined: R0: Risk to households with no water filter (they drink water from the tube well without any filtration), R1: Risk to households with water filters (until now), R2: Risk to households with water filters over five years, R3: Risk to households with water filter for more than 10 years, and R4: Risk to households with water filters over a lifetime.

### 2.3. Statistical Analysis and Modeling

Data were entered using Epidata 3.1 (“The EpiData Association”, Odense, Denmark), and analyzed in SPSS 17.0 (IBM, New York, NY, USA). All parameters were presented as means and standard deviations (SD). We used a probabilistic (stochastic) process to analyze exposure data and risk characterization. Therefore, all the parameters collected, such as arsenic concentration (both before and after sand filtration), exposure frequency and exposure duration were fit to identify the most relevant probabilistic density functions. Using these functions, Monte Carlo simulations with 10,000 permutations were used to model skin cancer risk distributions, using @ Risk 5.5 (Palisade Corporation, New York, NY, USA) integrated on Microsoft Excel 2007.

**Figure 2:**
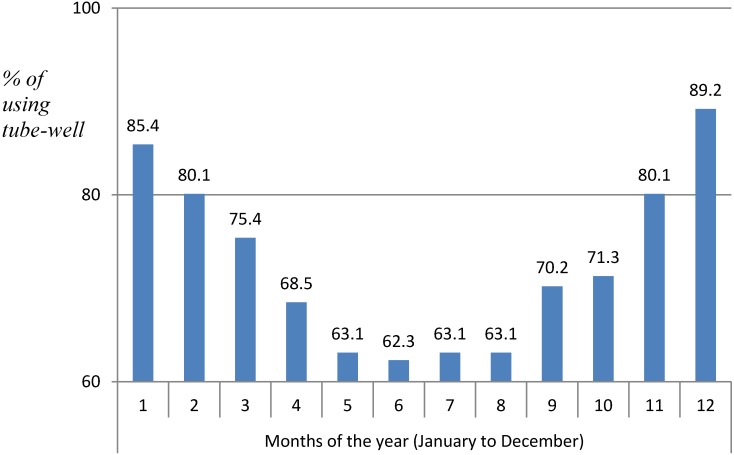
Status of tube-wells used for drinking purposes during one year.

## 3. Results

### 3.1. Issue Identification: Situation of Water Used for Drinking Purpose

In the 150 households participating in the study, 50% of the households used tube-well water for drinking (Group 1—full time using). The remaining 50% of the households used both well and rainwater for drinking purposes (Group 2—used them only part time). Rainwater was used mainly for drinking purposes, while well water was used for other purposes, such as bathing, washing food, and watering plants. Since the underground water in the studied commune also had very high levels of iron, the water has a strong metallic flavor. Therefore, all households in this area with water filters systematically use treated water for drinking and domestic purposes. The levels of water wells used for drinking purposes varied throughout the year, as shown in Figure 2.

People used water wells more in November, December, January and February (>80%). These were the dry months with very little rainfall. Meanwhile, the rates of using the water wells were lower in May, June, July, August (<65%), which were the summer months with plenty of rain. Local authorities and residents did express concern about the health risks related to high concentrations of arsenic in the drinking water samples taken from tube wells in different Communes in Ha Nam province in the past several years.

### 3.2. Hazard Identification and Dose-Response Assessment

#### 3.2.1. Hazard Identification

Arsenic (As) is a heavy metal that is found in inorganic and organic forms in drinking water, food, soil, dust, wood, and other materials [1,24,25]. Both organic and inorganic forms of As are present in varying amounts. Most of the As is in the organic form as arsenobetaine (AsB). Inorganic As presents in drinking water as either arsenate (As^5+^) or arsenite (As^3+^) [26]. Pentavalent (As^5+^) compounds are generally less toxic than trivalent (As^3+^) compounds, and the less water-soluble compounds are usually less toxic and less likely to have systemic effects than the more soluble compounds. Arsine gas (AsH_3_) is one of the most toxic inorganic arsenic compounds [1]. Food, such as fish and shellfish, contain relatively high concentrations of total As, with levels reaching into the parts per million range. Arsenic level as high as 19.3 mg/g dry weight was reported in oysters obtained from the Machu Islands of Taiwan [27]. Based primarily on studies of highly exposed populations in Taiwan and elsewhere, the U.S. Environmental Protection Agency (EPA) recently reduced the maximum contaminant level (MCL) standard for arsenic in drinking water from 50 µg/L to 10 µg/L [1]. The general population is exposed to As through drinking water, dust, fumes, and dietary sources, with the highest concentrations of As reported in seafood, rice, mushroom, and poultry [28]. Of the three possible routes of exposure to As (inhalation, ingestion, and dermal), ingestion is potentially the most significant source of exposure, and drinking water and food are the two primary ingestion pathways.

Inhalation of inorganic As was found to increase the risk of lung cancer and inorganic forms of As have been classified as human carcinogens [1]. The arsenic exposure time, which can lead to cancer, is 15–30 years of exposure, depending on the dose. Various studies in the past decades have shown increased risks of different types of cancer among people exposed to high levels of arsenic in the environment. Chronic As exposure in the range of 0.01–0.04 mg/kg/day has been reported to increase the incidence of skin cancer, lung cancer, bladder cancer, and other types of cancer in Taiwan [4,29]; respiratory cancers in Montana [30]; and bladder cancer in Finland [31]. Literature in the last few years also reported skin lesions commonly seen among Bangladesh subjects who ingested As contaminated drinking water [25]. In addition, chronic exposure with the concentration of arsenic in the dose range from 0.01–0.04 mg/kg/day increased the mortality rates due to heart disease, high blood pressure, and kidneys disease. Apart from the various adverse health effects that have been reported among people exposed to high levels of As, a growing number of laboratory studies, both in cell cultures and in experimental animals, have demonstrated the biologic effects of As at very low levels equivalent to those below the new 10 µg/L standard. These effects include endocrine disruption, altered cell signaling, altered cell cycle kinetics, alterations in proliferative response, and other effects, which may be associated with carcinogenesis and other disease processes [32]. Thus, it is important to understand the potential adverse effects of such exposure in the population.

#### 3.2.2. Arsenic Concentration in Tube-Well Water Before, after Sand Filtration and the Effect of Arsenic Removal

**Figure 3:**
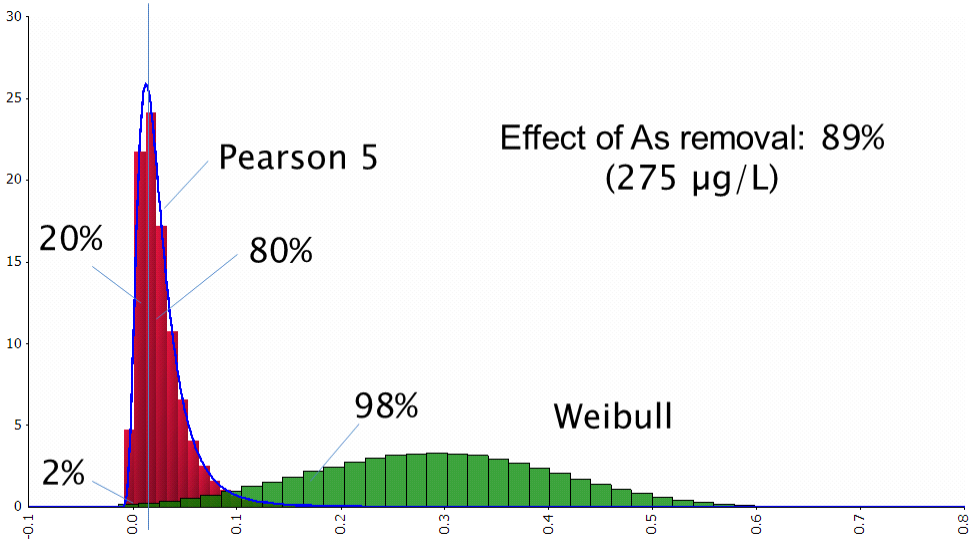
As concentrations in tube-well water before (green) and after (red) sand filtration.

The results showed that arsenic concentrations in tube-well water ranged from 8–579 ppb (mean 301 ppb) before filtration (Figure 1). All households used tube-well water for drinking purposes and used sand filter tanks to filter water before use. 95% of the survey households used self-designed filters or followed the designs of other households’ filters. Aeration systems increased the efficiency of the filters to remove arsenic, but only 9.5% of the households had these systems. 95.3% of the households had filters with thinner filtration media than the standard, and 66.9% of the filters did not meet the standard time for changing / back-washing the filter materials. Sand filters helped to remove 83% of As from water, however, since the concentrations of arsenic in the water sources were extremely high, the majority of the water samples after filtration still had As levels higher than the permitted level of 10 ppb (0.01 mg/L). Figure 3 shows the distribution of the arsenic quantitative results in water samples.

The concentrations of arsenic in water wells before filtration were represented by the Weibull function and up to 98% of the values exceeded the standard level (Figure 3, here in green). Pearson 5 function showed levels of arsenic in well water after filtration with 80% of the values exceeding the standard level (here in red).

#### 3.2.3. Dose-Response Assessment

By reviewing epidemiological data from five studies undertaken from 1968 to 2001, Tchounwou *et al*. (2004) reported that As exposure in drinking water in a strongly dose-dependent manner was correlated to both acute and chronic symptoms [33]. For example, 9%, 16%, and 44% incidence of As poisoning symptoms were reported at As concentrations in drinking water of 50, 50–100, and >100 µg/L, respectively [33]. For the chronic effects, strong dose-response correlations have been observed between As levels in drinking water and age-adjusted mortality rates for cancers of various organs, such as the lung, liver, bladder, kidney, and colon (Chen and Lin, 1994, cited in Tchounwou *et al*., 2004). There was very little data available to support a threshold for inorganic As carcinogenicity.

Tchounwou *et al*. (2004) also report data from the United States EPA (1980) that cancer risks of 10^−5^, 10^−6^ and 10^−7^ have been estimated for drinking water containing 0.022, 0.0022, and 0.00022 µg As/L [33]. These As levels, however, were approximately 450 times lower than the current As standard level for drinking water, which was set to be at 10 µg As/L for a lifetime cancer risk of 1 in 10,000 (U.S. EPA, 2006). If the data reported by Tchounwou *et al*. (2004) were accepted, then the current standard set by the U.S. EPA would result in unacceptable risk for a large population. This, however, is thought to be unlikely the case. Furthermore, one limitation of the dose-response assessment stage in this study is that the authors did not estimate the acceptable intake levels for As, such as the acceptable daily intake—ADI, or provisional tolerable weekly intakes—PTWI. The Provisional Tolerable Weekly Intakes for inorganic As recommended by the Joint FAO/WHO Expert Committee on Food Additives (JECFA) was 15 µg/kg bw/week [34]. More current data from U.S. EPA (1991) show that the Reference Dose for chronic oral exposures is 0.3 µg/kg/day, which is based on a NOAEL of 0.8 µg /kg/day and a LOAEL of 14 µg/kg/day for hyper-pigmentation, keratosis, and possible vascular complications in a human population consuming As-contaminated drinking water [20].

### 3.3. Exposure Assessment

In each household, the interviewee was the head of the household, either the wife or husband. Most of the interviewees (64.9%) have a secondary education level and 23.6% had a primary education level. A few people had a university degree, college or did not go to school, less than 2%. 74.0% of the interviewees were farmers and some of them were free labor (21.3%). Other occupations accounted for only 4.7%.

The estimated daily doses showed that up to 40% of the adults were affected from using arsenic contaminated water for drinking purposes as their daily intake exceeded the recommended TDI at1 µg/kg/day. Table 2 presents the most relevant distribution functions, which were fit to different parameters of the risk model.

**Table 2:** The parameters of the fitted distributions.

No	Parameter	Group 1		Group 2	
		Function	Parameter	Function	Parameter
1	C1	Weibull	RiskWeibull (3.5058, 0.40680, RiskShift (−0.077727))	Weibull	RiskWeibull (5.0414, 0.59058, RiskShift (−0.22853))
			M = 0.289		M = 0.314
			SD = 0.116		SD = 0.123
2	C2	Pearson5	M = 0.028	Pearson5	M = 0.0252
			SD = 0.0267		SD = 0.0214
			RiskPearson5(5.0000,0.18592, RiskShift (−0.018661))		RiskPearson5(6.4116,0.24312, RiskShift (−0.019758))
3	EF/30			NegBin	r = 3
					*p* = 0.46875
					M = 3.4
					SD = 2.693
					RiskNegBin (3,0.46875)
4	ED	NegBin	r = 6	NegBin	r = 5
			*p* = 0.356		*p* = 0.28
			M = 10.867		M = 12.854
			SD = 5.527		SD = 6.779
			RiskNegBin (6, 0.35573)		RiskNegBin (5, 0.28006)

Notes: C1: Concentration of As in tube-wells before filtration; C2: Concentration of As in tube-wells after filtration; EF × 12/365: Total months per year using tube-well water for drinking; ED: Total years using tube-well water for drinking. Group 1: using only As contaminated water, Group 2: using both wells and rainwater.

### 3.4. Risk Characterization

The average risk of skin cancer, calculated from 10,000 irritations of Monte Carlo simulations, caused by As contaminated consumption are presented in Table 3 with different scenarios.

**Table 3:** The results of estimating skin cancer risk (average risk from 10,000 permutations) for 2 groups based on the scenarios.

	Group 1	Group 2	Group 1/ Group 2
R0	262.0 × 10^−5^	96.4 × 10^−5^	2.72
R1	25.3 × 10^−5^	7.63 × 10^−5^	3.32
R2	36.8 × 10^−5^	10.8 × 10^−5^	3.41
R3	48.5 × 10^−5^	13.7 × 10^−5^	3.54
R4	405.0 × 10^·5^	104.0 × 10^−5^	3.89

Notes: R0: Risk with no filter; R1: Risk with filter (until now); R2: Risk with filter +5 years; R3: Risk with filter +10 years; R4: Risk with filter for lifetime; Group 1: Using As contaminated water only, Group 2: Using both well and rainwater.

**Figure 4:**
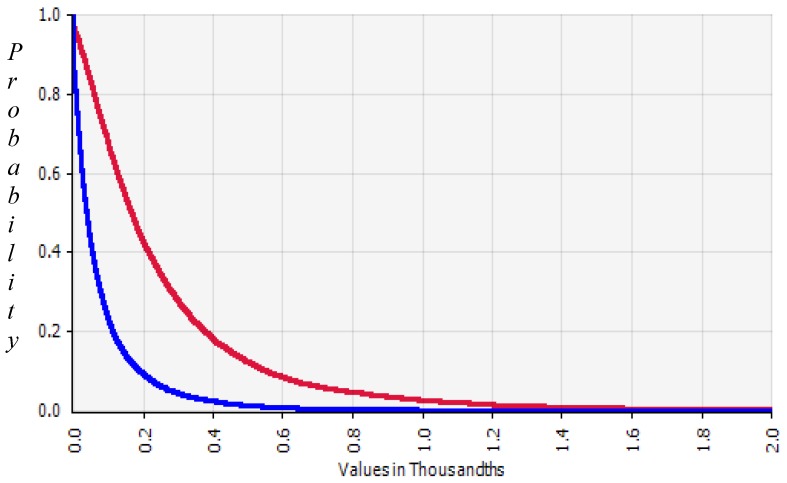
Skin cancer risk by using As contaminated water. Skin cancer risk with filter for Group 1 (red, R0) and Group 2 (blue, R1).

**Figure 5:**
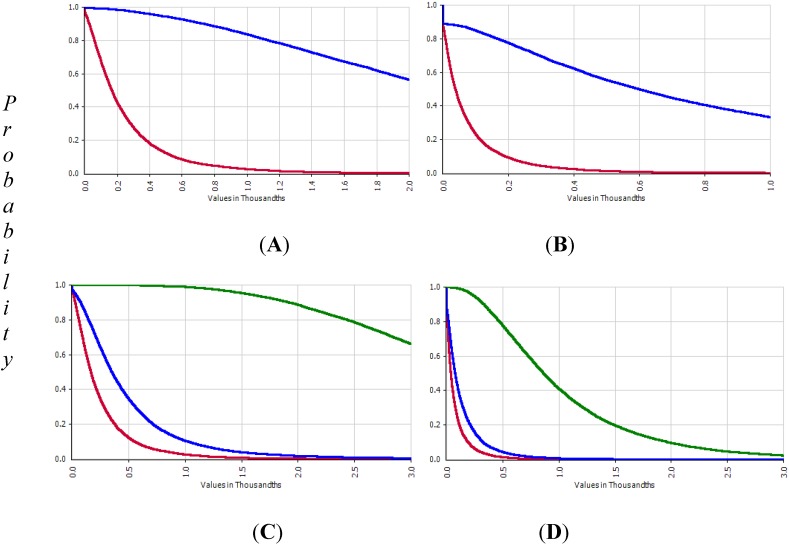
Skin cancer risk from using As contaminated water. Skin cancer risk with filter (red), +10 years (blue) and for lifetime (green) in Group 1 (**A**) and 2 (**B**) and without filter (blue) for Group 1 (**C**) and Group 2 (**D**).

The probability distribution of skin cancer risk, according to the scenarios, is shown in Figure 4 and Figure 5. The risk of the full-time using well water group (Group 1) was 3.32 times higher than the part-time group (Group 2). The risk of not using filtered water was 11.5 times higher than that with filtration. For the 5- or 10-year perspective from the time of the study (R1), the risk would increase 1.4 and 1.9 times compared to the currently estimated risk. If the people in Group 1 continued using the current arsenic contaminated water over the next 10 years or for a lifetime, the risk will increase by 1.9 and 16 times. If the people in Group 2 continue using the current arsenic contaminated water for more than 10 years or for a lifetime, the risk will increase by 1.8 and 13.6 times.

## 4. Discussion

The application of the Environmental Health Risk Assessment Framework helped to quantify the health risk due to exposure to arsenic in drinking water in an objective, systematic and participatory way. Most of previous studies reviewed describe arsenic toxicology and arsenic contamination in groundwater around the world and in Vietnam [12,14] and deal less with the direct health impact of As on the community. Therefore, the contribution of the research is the assessment of the level of exposure, time of exposure and average arsenic dose consumed. From these data, health effects and the risk of having cancer were quantified. Arsenic levels in water samples were measured quantitatively. This method was useful for achieving realizable data for analyzing the linear relationship between two or more quantitative variables and enables more accurate risk assessments. The risk assessment approach used in this study also encouraged the participation of the community and related stakeholders to ensure the quality of the assessment. Calculating the risk based on using a probabilistic approach captured more variability of the considered risk than the point estimate approach. With this approach, information on exposure and arsenic concentration in samples were described as distribution functions instead of average values. Exposure characteristics of the population were evaluated rather than calculating from data from individuals or from literature review. One of the limitations of this study is the lack of data on exposure levels to arsenic via food, air and skin contact. For example, there is evidence that inorganic arsenic is also present in food sources, such as rice. However, the study area selected was remote from industrial zones, thus, arsenic in the air was likely to be minimal. Moreover, arsenic contamination via food is proved to be of little risk because organic arsenic is much less toxic to humans. People in Chuyen Ngoai Commune who were using arsenic contaminated water were at high risk for skin cancer. If drinking water from wells was the only source of arsenic exposure, the average risk of cancer for adults were 25.3 × 10^−5^ (Group 1) and 7.6 × 10^−5^ (Group 2). The average lifetime risk was up to 405 × 10^−5^ (Group 1) and 104 × 10^−5^ (Group 2), much higher than the WHO Guidelines for Drinking-Water Quality, which has the recommended tolerable risk of 1 × 10^−5^ (for genotoxic carcinogens) [35].

If drinking water from wells was the only source of arsenic exposure, the average risk of cancer for adults in Chuyen Ngoai Commune was estimated at 25.3 × 10^−5^ (Group 1). It means that for every 100,000 adults in Chuyen Ngoai commune, 25 people will have cancer due to the consumption of water from wells after filtration. The average lifetime risk was up to 405 × 10^−5^. If compared to many other chemicals, the risk due to arsenic exposure was quite high. For example, the risk of cancer due to passive smoking range from 10 × 10^−5^ (low degree of exposure, *i.e.*, not married to smokers) to 120 × 10^−5^ with high exposure (married to a smoker). The risk of having cancer due to indoor radon (average concentration 50 Becquerel/m^3^) is 20 × 10^−5^ and that for benzene emissions in big cities (average concentration of 80 μg/m^3^) is 45 × 10^−5^ [19]. Water filtration in households helped to reduce 11.3-fold in the risk of cancer. The filtration effectiveness could be improved by small adjustments, such as installing aeration, increasing the thickness of the filter layer, replacing the filter material and washing filter materials periodically.

The uncertainty of this risk assessment was the daily amount consumption of arsenic that is calculated based on the average body weight of Vietnamese rural people. The volume of water consumed by an average adult used in this research was two liters (according to WHO guidelines). This would inevitably lead to errors due to the fact that the volumes of water consumed varied among individuals due to the physiological characteristics, seasons, *etc*. All samples were taken in one particular time. There was no longitudinal sampling to evaluate changes in arsenic concentrations in wells. The use of rainwater and water from wells for drinking purposes varied according to the seasonal variation in rainfall. Therefore, the accuracy of the exposure assessment would be affected. In addition, information used in the dose-response assessment and health hazard assessment for acute and chronic health impacts of arsenic was mainly secondary data from international studies. Furthermore, the responses of Vietnamese people to arsenic may differ to those of people worldwide. Moreover, the use of tube well As concentration is the least sensitive and specific arsenic exposure index measure and no biological sample, such as toenails or urine, were sampled to estimate As accumulation in the study subjects.

Risk assessment documents prove that people can get cancer if they drink arsenic-contaminated water. According to the World Health Organization (WHO), cancer has surpassed heart disease to become the leading cause of death worldwide. The rate of death from cancer in 2008 accounted for 13% and is equal to 7.6 million of the 57 million total deaths due to diseases worldwide. In the developed countries, approximately 25% of all deaths were due to cancers. In many developing countries, the cancer rate seems much lower, mostly due to higher mortality rates from infections and trauma. However, with the increasing control of infectious diseases, cancer rates have increased significantly [3]. According to the survey “Burden of disease and Injury in Vietnam—2008”, the main reasons for years of life lost were heart diseases (24%), cancers (21%) and unintentional injuries (17%). Although there is scientific evidence of the possibility of carcinogenicity due to arsenic, no particular types of cancer are reported to be closely associated with arsenic contamination. Skin cancer, kidney cancer and bladder cancer are classified as carcinomas, which are common types of cancer in chronic arsenic poisoning [36,37,38]. Arsenic is said to speed up the cell division, which leads to less windows (or leads to less time) for enzymes to repair DNA damage during DNA replication and increases the likelihood of genetic misleading. A mistake during the process of cell division can lead to cells receiving the wrong number of chromosomes (multiple chromosomes) and cause cancer. Endogenous carcinogenic factors are few and mainly are external factors (80%) [3]. Exposure to arsenic in the drinking water is an important external factor; therefore, preventing arsenic exposure will reduce the incidence of cancer.

Throughout the environmental health risk process, the research group has consulted a number of related stakeholders, including local authorities, the National Institute of Occupational and Environmental Health—Ministry of Health, the Centre for Environmental Technology and Sustainable Development (CETASD), the University of Science—National University, Hanam Preventive Medicine Center, the Division of Water Resources and Hydrometeorology, Hanam Department of the Environment and Natural Resources, the Health Environment Management Agency—Ministry of Health, the Department of Environmental Health—Hanoi School of Public Health, Chuyen Ngoai Commune’s People’s Committee, Duy Tien District Health Centre, Chuyen Ngoai Commune Health Centre and the local community. Representatives from stakeholders showed interests and had important opinions, as well as providing valuable materials and information throughout the risk assessment process. A consultation workshop was also held at the local health center with the participation of representatives from the local authorities and health staff to discuss the results of the risk assessment, their opinions on the issue and recommendations on how to manage the risk. For risk communication activities, it should be noted that not all of the households were exposed to arsenic in drinking water with high concentrations: this happened only to the households using unfiltered or inappropriately filtered groundwater for drinking purposes. Building the aeration system, increasing the thickness of the filter bed, and following the filter design guidelines of the Vietnamese Ministry of Health proved to be efficient at removing arsenic from the groundwater.

## 5. Conclusions

The results showed that tube-well water in Chuyen Ngoai Commune was heavily contaminated with arsenic. The arsenic concentrations in drinking water before filtration were from 8 ppb to 579 ppb (the mean was 301 ppb). The majority of households designed their sand filters themselves, but these filters did not meet the standard for arsenic removal. The arsenic daily consumption of 40% of the adults was higher than the level of the TDI (1 µg/kg/day). The average skin cancer risks in adults due to consuming filtered tube-well water for drinking purpose were 25.3 × 10^−5^ (using only well water) and 7.6 × 10^−5^ (using both well and rain water). These skin cancer risk would be 11.5 times higher if the water was not filtered, and 1.9, 14.8 times higher if people continued using the current arsenic contaminated water for 10 more years or for a lifetime, respectively. Improved filtration measures or replacing the current drinking water sources (*i.e.*, with rain water or clean pipe water) to minimize the health risks to the local population were recommended.
